# CRISPR-Cas13a-powered electrochemical biosensor for the detection of the L452R mutation in clinical samples of SARS-CoV-2 variants

**DOI:** 10.1186/s12951-023-01903-5

**Published:** 2023-04-29

**Authors:** Zhi Chen, Chenshuo Wu, Yuxuan Yuan, Zhongjian Xie, Tianzhong Li, Hao Huang, Shuang Li, Jiefeng Deng, Huiling Lin, Zhe Shi, Chaozhou Li, Yabin Hao, Yuxuan Tang, Yuehua You, Omar A. Al-Hartomy, Swelm Wageh, Abdullah G. Al-Sehemi, Ruitao Lu, Ling Zhang, Xuechun Lin, Yaqing He, Guojun Zhao, Defa Li, Han Zhang

**Affiliations:** 1 Qingyuan People’s Hospital, The Sixth Affiliated Hospital of Guangzhou Medical University, Qingyuan, Guangdong 511518 People’s Republic of China; 2 International Collaborative Laboratory of 2D, Materials for Optoelectronics Science and Technology of Ministry of Education, Institute of Microscale Optoelectronics, College of Physics and Optoelectronic Engineering, Shenzhen University, Shenzhen, 518060 People’s Republic of China; 3 Yangtze Delta Region Institute (Huzhou), University of Electronic Science and Technology of China, Huzhou, 313001 People’s Republic of China; 4 Institute of Pediatrics, Shenzhen Children’s Hospital, Institute of Pediatrics, Shenzhen Children’s Hospital, Clinical Medical College of Southern University of Science and Technology, Shenzhen, Guangdong 518038 P. R. China; 5 College of Pharmacy, Dali University, Dali, 671000 P. R. China; 6 Hengyang Medical College, University of South China, Hengyang, Hunan 421001 China; 7 School of Physics & New Energy, Xuzhou University of Technology, Xuzhou, Jiangsu 221018 People’s Republic of China; 8 Shenzhen Metasensing Tech Limited Company, Shenzhen, 518000 People’s Republic of China; 9 Department of Stomatology, Longhua People’s Hospital Affiliated to Southern Medical University, Shenzhen, 518109 People’s Republic of China; 10 School of Stomatology, Southern Medical University, Guangzhou, 510515 People’s Republic of China; 11 Department of Physics, Faculty of Science, King Abdulaziz University, Jeddah, 21589 Saudi Arabia; 12 Research Center for Advanced Materials Science (RCAMS), King Khalid University, P.O. Box 9004, Abha, 61413 Saudi Arabia; 13 Department of Chemistry, College of Science, King Khalid University, P.O. Box 9004, Abha, 61413 Saudi Arabia; 14 Key Lab of Semiconductor Materials Science, Institute of Semiconductors, Chinese Academy of Sciences, Beijing, 100083 China; 15 Laboratory of All-Solid-State Light Sources, Institute of Semiconductors, Chinese Academy of Sciences, Beijing, 100083 China; 16 Shenzhen Center for Disease Control and Prevention, Shenzhen, Guangdong 518055 P. R. China; 17 Department of Laboratory Medicine, Shenzhen Children’s Hospital, Shenzhen, Guangdong 518038 People’s Republic of China; 18 Shenzhen International Institute for Biomedical Research, Shenzhen, 518110 China

**Keywords:** CRISPR, SARS-CoV-2 BA.5 variant, Electrochemical biosensor, MXene

## Abstract

Since the end of 2019, a highly contagious disease caused by severe acute respiratory syndrome coronavirus 2 (SARS-CoV-2) has deprived numerous lives worldwide, called COVID-19. Up to date, omicron is the latest variant of concern, and BA.5 is replacing the BA.2 variant to become the main subtype rampaging worldwide. These subtypes harbor an L452R mutation, which increases their transmissibility among vaccinated people. Current methods for identifying SARS-CoV-2 variants are mainly based on polymerase chain reaction (PCR) followed by gene sequencing, making time-consuming processes and expensive instrumentation indispensable. In this study, we developed a rapid and ultrasensitive electrochemical biosensor to achieve the goals of high sensitivity, the ability of distinguishing the variants, and the direct detection of RNAs from viruses simultaneously. We used electrodes made of MXene-AuNP (gold nanoparticle) composites for improved sensitivity and the CRISPR/Cas13a system for high specificity in detecting the single-base L452R mutation in RNAs and clinical samples. Our biosensor will be an excellent supplement to the RT-qPCR method enabling the early diagnosis and quick distinguishment of SARS-CoV-2 Omicron BA.5 and BA.2 variants and more potential variants that might arise in the future.

**Supplementary Information:**

The online version contains supplementary material available at 10.1186/s12951-023-01903-5.

## Background

The evolution of the severe acute respiratory syndrome coronavirus 2 (SARS-CoV-2) led to various variants. Omicron, the latest SARS-CoV-2 variant, was first reported in South Africa at the end of 2021 and spread out to many countries. Furthermore, five sub-lineages can be derived from the Omicron lineage (B.1.1.529), named BA.1 to BA.5 [1]. Omicron variants spread quickly among fully vaccinated and/or previously infected individuals because of the increased transmissibility and antigenic shift [2]. During the period of this study, BA.2 and BA.5 are the two main subtypes rampaging worldwide, because BA.3 does not meet the “Nextstrain clade definition criteria.“ BA.4 and BA.5 resist most broad neutralizing antibodies because of their multiple mutated sites and the mutation at the spike amino acid 452 [3]. The L452R mutation likely affects the human angiotensin-converting enzyme-2 (hACE2) to facilitate antibody binding. On the other hand, an increased receptor binding affinity and in vitro infectivity are also why BA.5 has a stronger infection [4–6]. Therefore, the L452R mutation is an essential marker for detecting SARS-CoV-2 Omicron BA. 5 infections among people and monitoring the evolution of variants. Unfortunately, the current detection methods for SARS-CoV-2 are primarily based on polymerase chain reaction (PCR) and require further sequencing to determine the genome of the causative viral strain, usually taking 48 h [7].

The CRISPR-Cas system has been applied broadly as an innovative gene-editing technique [8]. Cas12 and Cas13, as two types of CRISPR/Cas nucleases, possess additional trans-cleavage activities after activation [9, 10]. This property has been successfully exploited in Point of Care Testing (PoCT) detectors for SARS-CoV-2 nucleic acid [11]. The Cas12a-CRISPR RNA (Cas12a-crRNA) complex can be activated by double-strand DNA (dsDNA) with desired gene sequence to generate fluorescence signals by trans-cleaving unspecific single-stranded DNA (ssDNA) fluorescence-quencher reporters. In contrast, Cas13a targets RNAs and trans-cleaves unspecific single-strand DNA (ssDNA) reporters [12, 13]. Previously, we developed MoPCS (Methodologies of Photonic CRISPR Sensing), a low-cost, CRISPR/Cas12a-based SPR gene-detecting platform to analyze SARS-COV-2 variants [14]. However, the CRISPR/Cas12a system is limited by reliance on a particular protospacer adjacent motif (PAM) in the target sequence [15]. There is usually no PAM sequence “coincidentally” near the single nucleotide polymorphism (SNP) sites that indicate variants of SARS-COV-2 [16]. For example, when testing for L452R mutation in a sample, a “TTTV” sequence is not found near this CRISPR/Cas12a system mutation site. Therefore, we mainly applied Cas13a instead of Cas12a because it does not require the PAM sequence, making it more versatile [12, 14, 17]. Cas13a has been applied to detect nucleic acids from viruses using fluorometry directly and lateral flow readouts [18]; now, it is a potential system to be developed and applied for electrochemical sensors. Another reason we applied Cas13a is that SARS-CoV-2 is an RNA virus [19] and Cas13a can directly detect extracted RNA without the processes for reverse transcription and pre-amplification, which could affect the accuracy and stability of the techniques [20, 21]. The major advantage of using the CRISPR/Cas13a system for the detection of SARS-CoV-2 directly is its high sensitivity. This study focuses on building an electrochemical technique combined with the CRISPR/Cas13a detection platform to improve the sensitivity of SARS-CoV-2 detection.

Electrochemical techniques offer promising applications using low-cost and easy-to-handle portable devices, especially for medical diagnostics and environmental monitoring. At the same time, the development of novel 2D materials (graphene, MXene, etc.) and their related hybrids have improved the sensitivity and stability of electrochemical detection of SARS-CoV-2 [22–24]. Electrochemical biosensors combine the high sensitivity of the sensors and the specificity of biomolecule recognition strategies. Therefore, combining CRISPR-Cas with electrochemical techniques and advanced 2D materials has excellent potential in nucleic acid-related diagnostics [25, 26]. A new group of functional 2D transition metal carbides/nitrides, MXenes, are increasingly applied due to their unique properties, including metal conductivity, surface hydrophilicity, and large chemically active surfaces, which are promising for electrochemical applications [27]. DNA sensing broadly follows two formats by taking advantage of MXenes by (a) directly attaching DNA/RNA (labeled or non-labeled) to MXenes via π–π bonds or (b) modifying MXene surfaces with polymers, nanoparticles, etc., such groups or sites facilitate DNA/RNA binding with MXenes. The most widely utilized nanoparticles for achieving this goal are gold nanoparticles (AuNPs) with excellent biocompatibility, which usually offer attach sites for thiol-modified DNA/RNA through a stable S-Au interaction [28]. We have recently proposed the methodology of electrochemical CRISPR sensing (MoECS) for detecting the SARS-COV-2 delta variant [29]. This previous system combines electrochemical technology and CRISPR-Cas cleavage and has potential applications in PoCT for SARS-CoV-2 variants screening conveniently.

In this study, we further improved the “MoECS” system to detect Omicron BA.5 variants by introducing Cas 13 and designing new crRNAs targeting Omicron variants. To enhance sensing performance, the working electrode (AuE) was first functionalized with MXene-AuNP composites (AuE-MXene-AuNPs) to improve its electrochemical surface area and conductivity. It was then covered with thiol- and methylene blue-modified single-stranded RNA (SH- ssRNA-MB), which acts as the reporter gene by forming an S–Au bond. When the fabricated system is treated with the Cas13a-crRNA-target RNA reaction matrix, Cas13a is activated, resulting in the non-specific trans-cleavage of the ssRNA-MB from the AuE-MXene-AuNPs surface. Therefore, under the trans-cleavage by CRISPR/Cas13 system, electron transfer between the electrode and the redox mediator (MB) connected by the ssRNA would be changed, and this variation can be transduced by an electrochemical platform and detected. The advantages of building an electrochemical biosensor based on MoECS for distinguishing SARS-COV-2 Omicron BA.2 and BA.5 variants were tested and reflected through biological and electrochemical experiments. Moreover, the limit of detection (LoD), stability, and specificity of the biosensor was further verified. Finally, the clinical samples identified as BA.2 or BA.5 collected from a period of a local outbreak in Shenzhen, China, had been tested with this platform, and the accurate variant was reported while the samples were defined as “positive”. Herein, this novel CRISPR/Cas13a based electrochemical detection platform can offer stable, precise, and sensitive gene analysis of clinical samples with BA.2 or BA.5 variants, and it could be further extended to the test of emerging SARS-Cov-2 variants in the future.

## Results and discussion

**Fig. 1:**
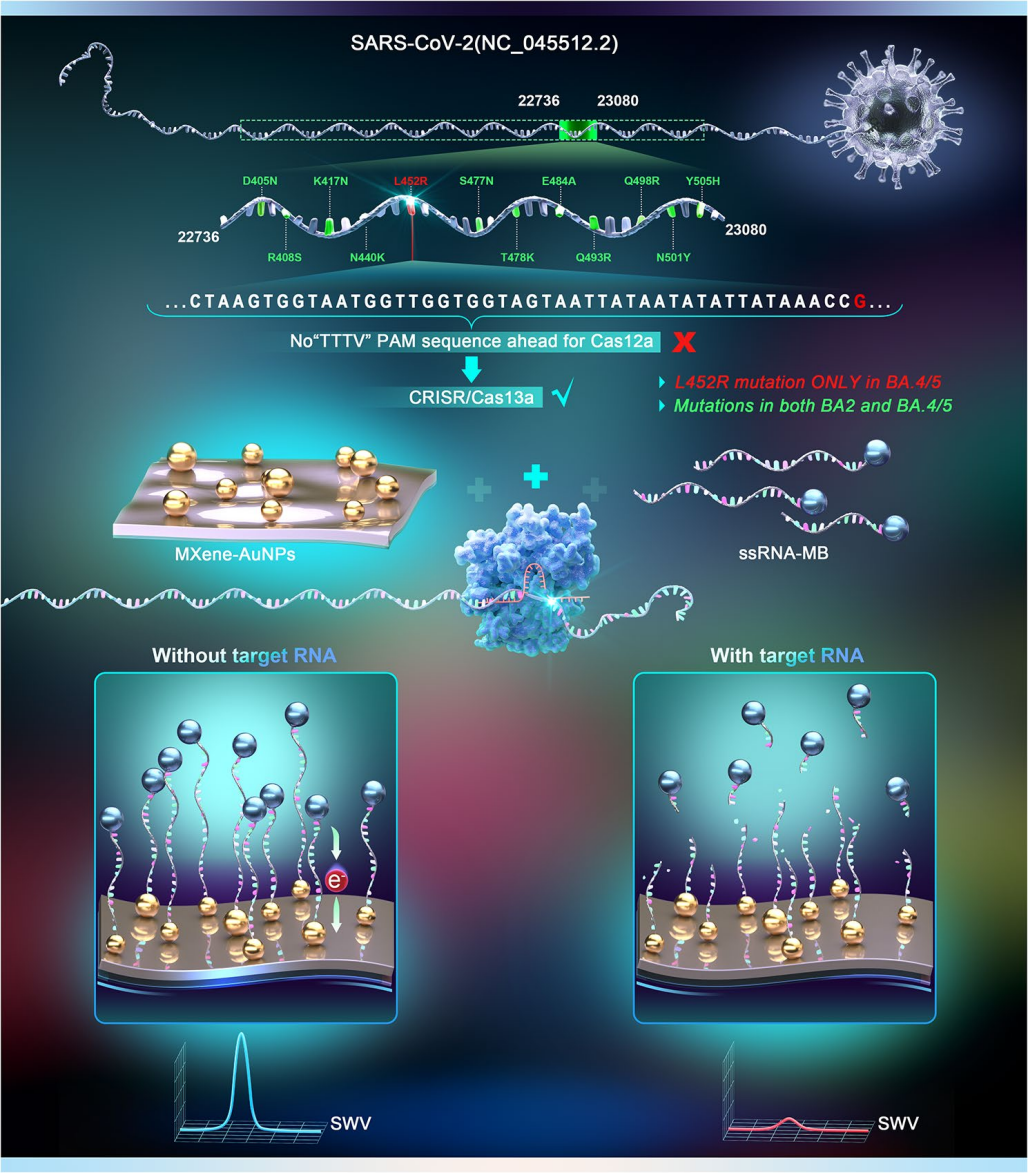
A schematic illustration of SARS-COV-2 nucleic acid detection based on MoECS using the CRISPR-Cas13a system (MoECS: methodology of electrochemical CRISPR sensing)

The mechanism of SARS-COV-2 Omicron BA. 5 variant detection based on electron transfer between MXene-AuNPs and methylene blue on the ssRNA-MB in the CRISPR-Cas13a system is illustrated in Fig. 1. The Omicron variants harbor multiple mutations in the S gene of the SARS-COV-2 virus. The Omicron BA.2 and BA. 5 variants share many of these mutations (such as D405N and R408S, labeled in green). However, the BA. 5 variants have the featured L452R mutation, which was selected as the target detection site. The collateral cleavage property of the CRISPR/Cas13a system was utilized for the flexibility and specificity of detection. The stable S-Au bonds on the MXene-AuNPs modified electrodes lead to SH-ssRNA-MB adsorption, and the electrode is treated with a CRISPR mix solution (customized crRNA, Cas 13a, and target RNA) [17, 30]. In the absence of target RNA, both the specific cis-cleavage activity and the non-specific trans-cleavage activity of Cas13a are not activated [31]. While the ssRNA-MB was still adsorbed on the electrode surface after being treated with the CRISPR mix solution, the MB redox on the ssRNA contributed to a distinct current signal. In the presence of the target RNA, the cis- and trans-cleavage activities of CRISPR-Cas13a are activated, cleaving the ssRNA reporters nonspecifically from the electrode surface and significantly reducing the corresponding redox current signal of MB. While a change in the redox current indicates the presence of target RNA, the magnitude of the decrease in the electrochemical signal quantifies the target RNA concentration [32, 33].

**Fig. 2:**
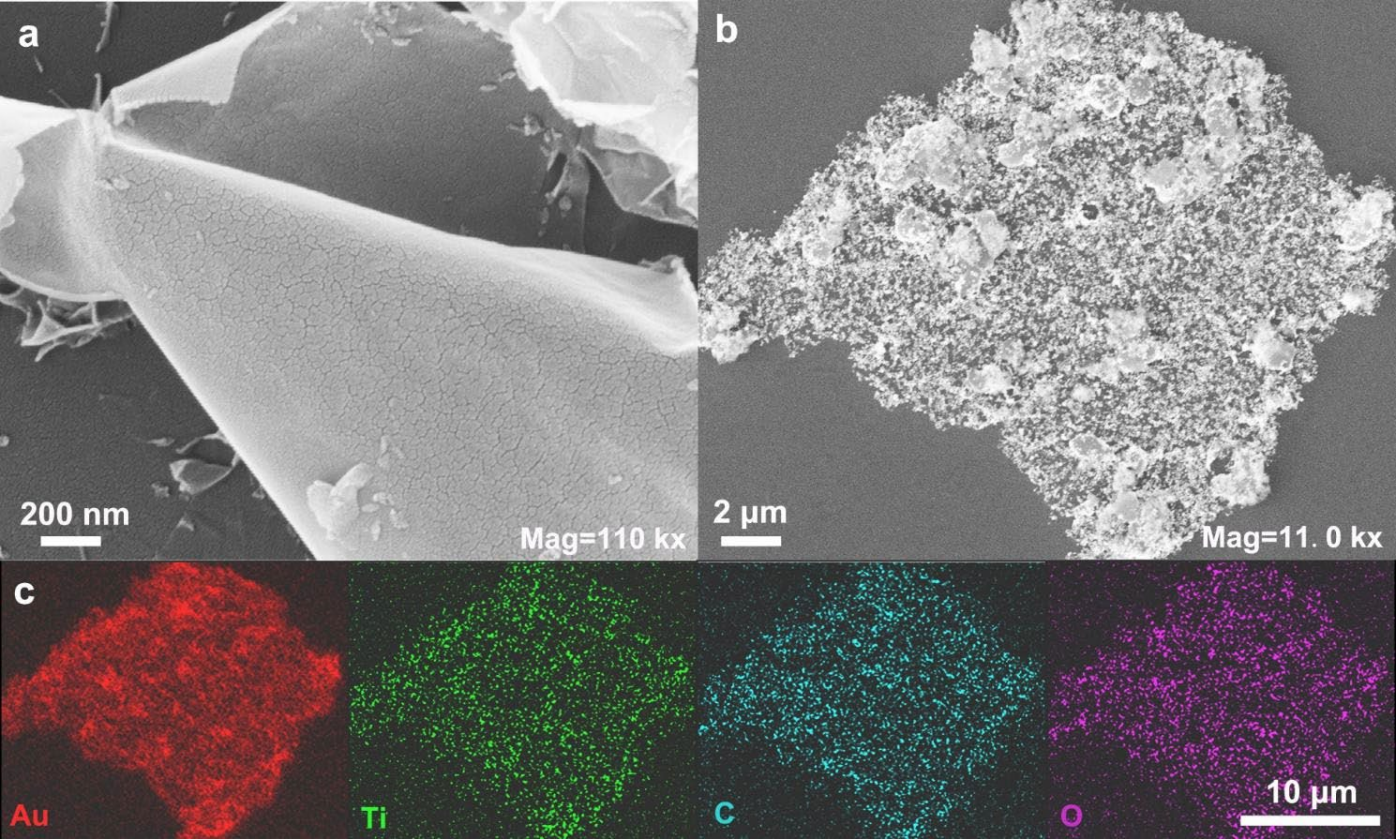
Scanning electron microscopy (SEM) Shown are the SEM images of (a) ultrathin Ti_3_C_2_ nanosheets (MXene) and (b) MXene-AuNPs composites. (c) SEM-EDS images of corresponding elemental mapping of Au, Ti, C, and O for MXene-AuNPs (AuNP, gold nanoparticle; EDS, energy-dispersive X-ray spectrometry)

We used an ultrathin Ti_3_C_2_ nanosheet as a reductant for preparing MXene-AuNPs and as a substrate for supporting the AuNPs. The successful preparation of HF-etched Ti_3_C_2_ nanosheets was the first step in building the electrochemical sensing platform. As shown in Fig. 2a, ultrathin single-layered flat Ti_3_C_2_ nanosheets were formed because the weakened interlayer interactions facilitated the formation of monodisperse nanosheets after DMSO intercalation and ultrasonic mechanical delamination treatments [34]. In addition, XRD patterns of Ti_3_AlC_2_ powder and the etched Ti_3_C_2_ nanosheets were generated (Figure S1). After selective etching, the distinct peak at 39.1° corresponding to the (104) plane of Ti_3_AlC_2_ was significantly weakened, while the (002) peak dropped from 9.5° to 6.9°, indicating the removal of Al layers and successful preparation of Ti_3_C_2_ MXenes [27, 35].

In HAuCl_4_-citrate solution, Au^3+^ could be mildly reduced to Au^+^ by citrate at room temperature (25 °C), wherein the citrate ions act as reductants and stabilizers [36–38]. As a result, the subsequent reduction of Au^+^ to AuNPs by MXene was easily activated. In other words, HAuCl_4_ pretreated with citrate protected MXene from excess oxidation to some degree. After adding the HAuCl_4_-citrate solution, the MXene nanosheets were partially oxidized while simultaneously forming the AuNPs, and several small particles were distributed on the surface of the nanosheets (Fig. 2b and Figure S2). In addition, elemental mapping of MXene-AuNP composites at the microstructural level was carried out by scanning electron microscopy (SEM) with energy-dispersive X-ray spectrometry (EDS). As shown in Fig. 2c, the homogeneous distributions of Au, Ti, C, and O over the entire MXene architecture confirmed that the small particles distributed on the Ti_3_C_2_ nanosheets were in situ reduced AuNPs. Some studies have revealed that the electrochemical performance of controlled oxidized Ti_3_C_2_ MXenes was improved, and new applications of partially oxidized MXenes have been extended to energy and catalysis [39, 40]. Thus, we expected the hybrids of partially oxidized MXene and AuNPs to work. The (Electrochemical Impedance Spectroscopy) EIS of pristine MXene nanosheets and MXene-AuNPs was performed to evaluate the interfacial electron transfer efficiency [41]. As expected, the electron transfer resistance (R_et_) of partially oxidized MXene-AuNPs was approximately 50 Ω, which was much smaller than that of the MXene nanosheets (150 Ω), indicating the high conductivity of partially oxidized MXene-AuNPs (Figure S3). Besides, the cyclic voltammetry (CV) of MXene-AuNPs composites in 0.5 M KOH showed a distinct redox peak of Au at 0.1 V vs. Ag/AgCl compared to pristine MXene, which further verified the formation of AuNPs (Figure S4) [42, 43].

The CRISPR/Cas system design was a crucial part of this study to ensure high specificity in detecting BA. 5 variants. As shown in Fig. 1, a sequence containing various mutation sites on the S gene of SARS-CoV-2 (NC.045512.2, 22,736–23,080) was selected as the RNA template (Table S2). Among these mutations, L452R is found in BA. 5 variants but not in BA.2 variants (the main variant found in patients in China). Therefore, L452R was selected as the target mutation site to detect and distinguish between the BA. 5 and BA.2 variants. However, since there is no “TTTV” PAM sequence adjacent to the L452R mutation, Cas12a, the CRISPR/Cas protein used in the E-CRISPR technique, cannot bind to this region. Alternatively, we utilized the CRISPR/Cas13a system because it has no PAM sequence requirements, offering high flexibility to target SNP sites.

Cis- and trans-cleavage are the core features of the CRISPR/Cas13a system when detecting target sequences. Lane 1 in Figure S5 shows an intact wild-type SARS-CoV-2 RNA template, while lane 2 shows the same template after reacting with a CRISPR matrix containing a crRNA targeting the original SARS-CoV-2 sequence (crRNA-ori) and a fluorescence-quencher reporter. As seen in lane 2, the RNA band was spliced into two, indicating successful cis-cleavage. The upper band appears to be greater in size than the original template, which could be because part of the template was still bound to the Cas13a protein after cis-cleavage and, therefore, lagged during electrophoresis. In contrast, the lower band was probably freed from the Cas13a protein and moved faster than the original template because of its smaller size. The lowest band showed strong green FAM fluorescence, indicative of trans-cleavage and splicing of the F-Q reporter. Lane 3, with no template RNA, shows no FAM fluorescence.

**Fig. 3:**
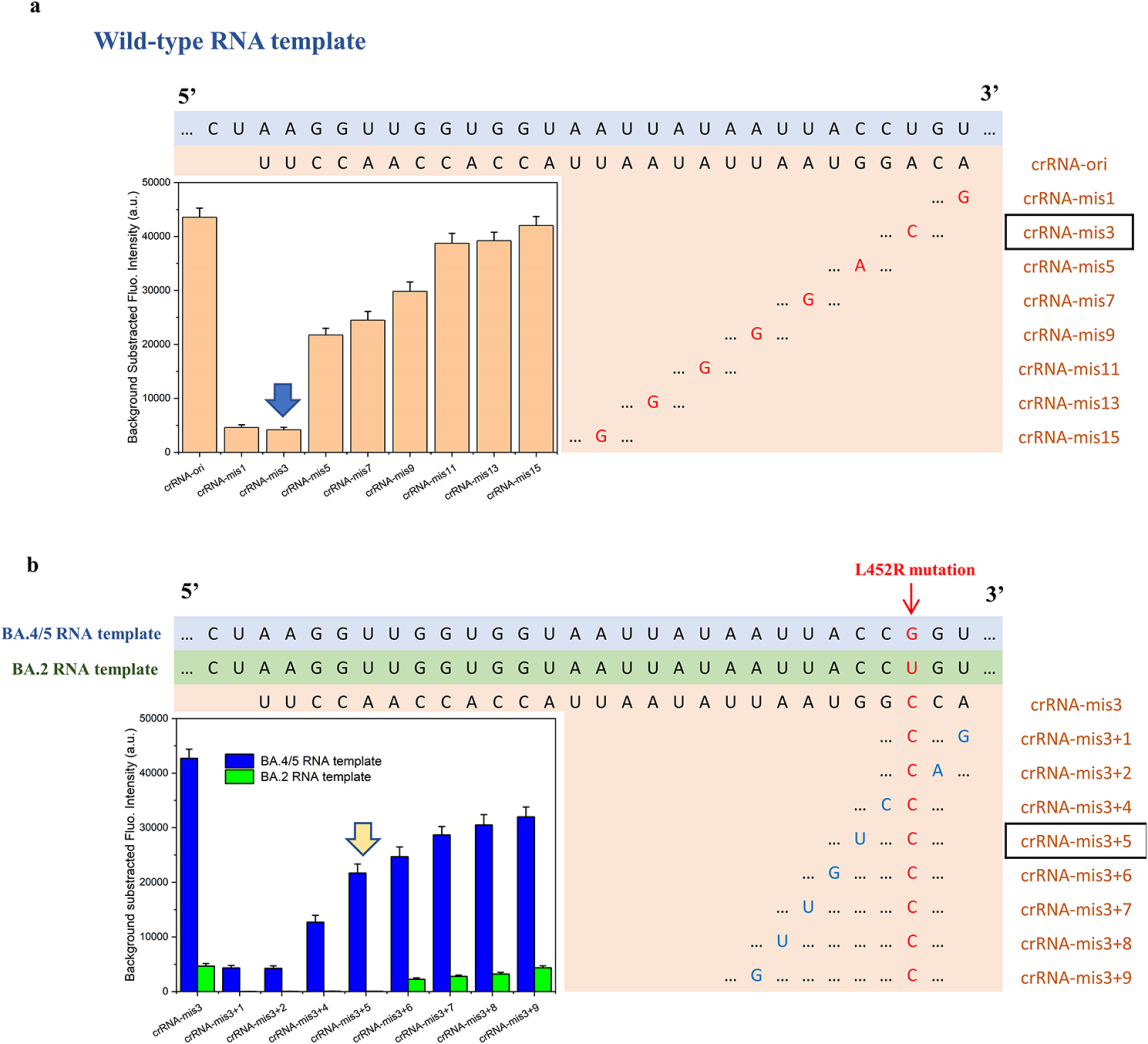
Optimization of crRNAs Fluorescence analysis of crRNAs introduced with (a) a single mismatch at different sites targeting the SARS-CoV-2 wild-type RNA template and (b) a dual mismatch at different sites targeting the BA.2 and BA. 5 RNA templates (crRNA, CRISPR RNA)

The Cas13a protein acts like a pair of “scissors” cleaving the RNA sequence. The crRNAs bind to Cas13a to form a Cas13a-crRNA complex, which acts as “smart scissors” to cleave the desired target RNA sequence and spare the “innocents” even if one single base is wrong. The delicate design and crRNA selection are vital for the specificity of the CRISPR/Cas13a system. We tested the binding of crRNAs with mismatches at different sites to wild-type SARS-CoV-2 RNA templates at 1 nM concentration (Fig. 3a). A mismatch in the 1st or 3rd base of the protospacer (crRNA-mis1 and crRNA-mis3) dramatically decreased the fluorescence but was still not negligible (10.47% and 9.51%, respectively). As the mismatch site became more distant, the decrease in fluorescence became increasingly weaker; the effect of crRNA-mis11-15 was comparable to crRNA-ori. A mismatch at the 3rd site of the protospacer resulted in the lowest trans-cleavage activity. Therefore, the SNP site (L452R mutation) can be placed on the 3rd site of the protospacer of the crRNA to achieve the most significant difference between on-target and off-target samples. Nonetheless, the off-target trans-cleavage activity influences the accuracy of L452R mutation detection.

To resolve this issue, a “dual mismatch” was introduced in the next step (Fig. 3b). For the BA. 5 RNA template, crRNA-mis3 was the “right” crRNA. The BA.2 RNA template was considered similar to the “wild-type” sequence because there were no mutation sites in this range. An additional mismatch in the 1st − 9th site of the protospacer (“crRNA-mis3 + 1” to “crRNA-mis3 + 9”) was introduced, and each of the resultant crRNAs was allowed to bind with the BA.2 and BA. 5 RNA templates. When the additional mismatch site was located on the 1st − 5th site of the protospacer, no fluorescence signal was detected when tested with the BA.2 template, while mismatch on the 5th site still generated relatively high fluorescence when tested with the BA. 5 template (50.83% of the perfect match). Therefore, crRNA-mis3 + 5 was selected as the optimal crRNA targeting BA. 5 RNA sequences with the ability to distinguish single-nucleotide changes. After designing the CRISPR/Cas13a system for RNA sequence specificity, an electrochemical platform was built for sensitivity in the following steps.

**Fig. 4:**
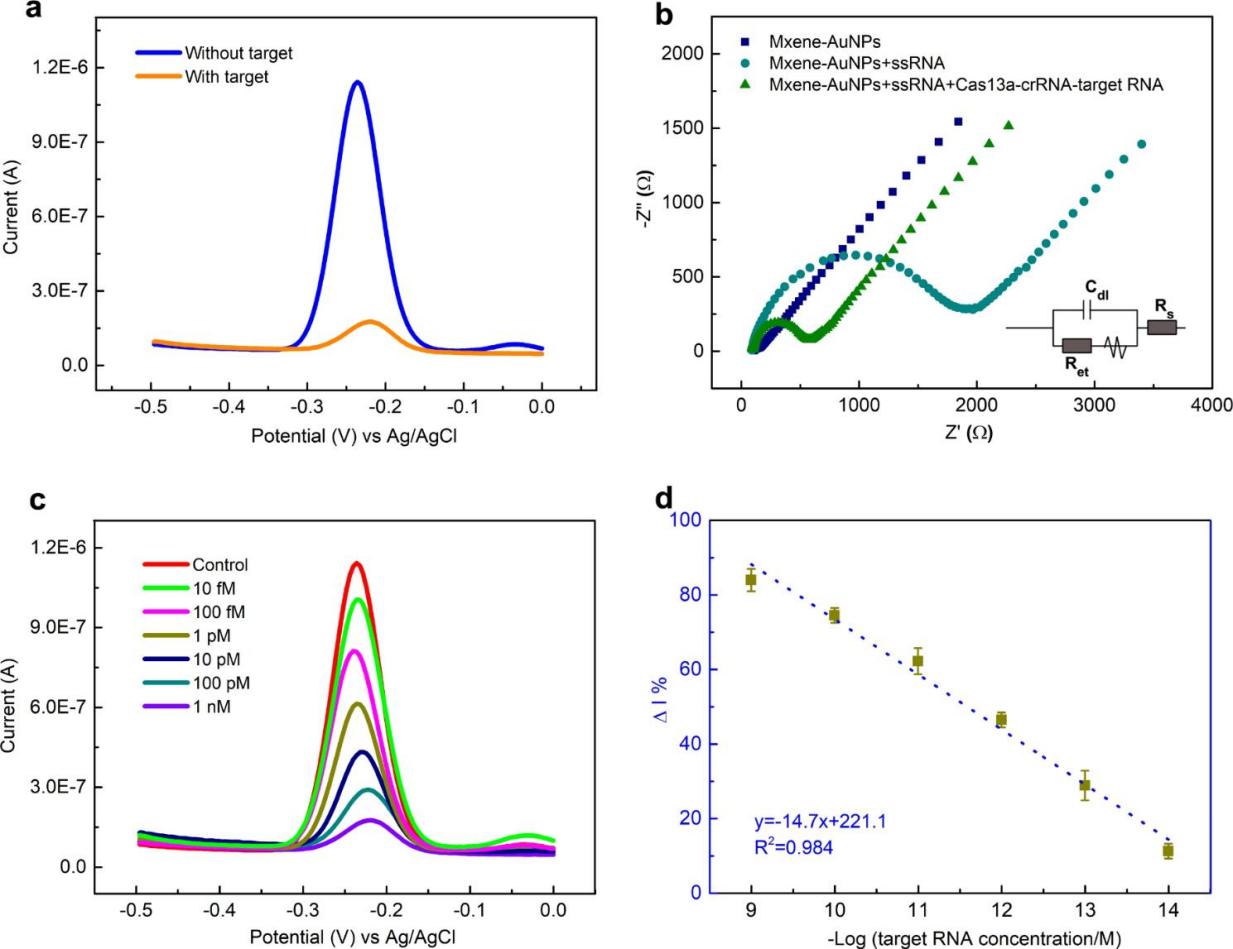
Evaluation the electrochemical performance of fabricated biosensor **(a)** Square wave voltammetry (SWV) curves for with (1 nM) and without target RNA. **(b)** EIS performs the preparation and reaction stages of the biosensor. **(c)** SWV curves for target RNA concentrations ranging from 1 nM to 10 fM. Control refers to the replacement of target RNA with pure H_2_O. **(d)** The linear relationship between the change of current (Δ I%) and the -logarithm of the target RNA concentration. Error bars represent standard derivations obtained in three parallel experiments

The CRISPR-Cas13a-based biosensor was analyzed using electrochemical techniques to explore the cleavage by MXene-AuNP-modified Au electrode. After the mix of target RNA and Cas13a-crRNA complex, Cas13a-crRNA-target RNA triplex was incubated with the ssRNA-MB-decorated electrode directly. We first employed SWV technique to evaluate the MB signal changes after the triplex complex treatment. As shown in Fig. 4a, in the presence of the triplex complex, the MB signal was significantly decreased (the gray curve) compared with the background signal without the target (the green curve). In addition, EIS was selected to perform the electrochemical properties of the MXene-AuNP-modified electrode at different conditions (Fig. 4b) [33, 44]. For the bare MXene-AuNP-modified electrode, the Nyquist plot was almost a straight line, and only a small semicircle appeared in the high-frequency region, indicating an ultra-small R_et_. When the electrode was covered by the ssRNA-MB reporter, the detected R_et_ increased to 1800 Ω, which could be attributed to the electrostatic repulsive force with [Fe(CN)_6_]^3−/4−^ caused by the self-assembled negatively charged ssRNA-MB monolayer [44]. After adding the triplex complex on the ssRNA-MB-modified electrode, the detected R_et_ decreased to 600 Ω. Variations in both the SWV signal and EIS indicated that ssRNA-MB reporters could be successfully trans-cleaved and then separated from the electrode surface after treatment with the triplex complex. In summary, these results demonstrated that the CRISPR-Cas13a-based electrochemical biosensor could be used for nucleic acid detection.

In the CRISPR-Cas13a-based electrochemical biosensor, the trans-cleavage activity of Cas13a contributed to the amplification of the signal. To characterize the sensitivity of the biosensor, various concentrations (1 nM to 10 fM) of the Omicron BA4/5 target RNA were measured under optimal conditions. The redox current of the MB gradually decreased as the target RNA concentration increased as shown in Fig. 4c. Detection was rapidly completed within 1 h of treating the ssRNA-modified electrode with the CRISPR mix. Fortunately, the MB signal variation was linearly related to the log target RNA concentration, which was mapped using the correlation equation y = 221.1-14.7x. Furthermore, R^2^ was calculated to be 0.984 (Fig. 4d). A background (B) experiment was conducted without the target RNA, wherein the change in the mean current of the background (n = 3) was divided by three standard deviations (SD). B/3SD was calculated and used as the limit of detection (LOD), which was calculated to be 1 fM. Compared with our previous work (electrodes were modified by AuNPs only) [29], the LOD was dramatically decreased in this study, which could be ascribed to the improved conductivity and electrochemical surface area of the MXene-AuNP composites. Furthermore, the LOD of the electrochemical biosensor and the Cas13a-based fluorescence sensor were compared. An ssRNA-FQ probe was used as a reporter for the Cas13a-based fluorescence detection. The detection can be completed within 2 h, and the results are shown in Figure S6, in which the LOD of Cas13a-based fluorescence assay is 1.72 pM. Therefore, CRISPR-Cas13a-based electrochemical biosensors can potentially improve sensitivity by more than 1700-fold.

**Fig. 5:**
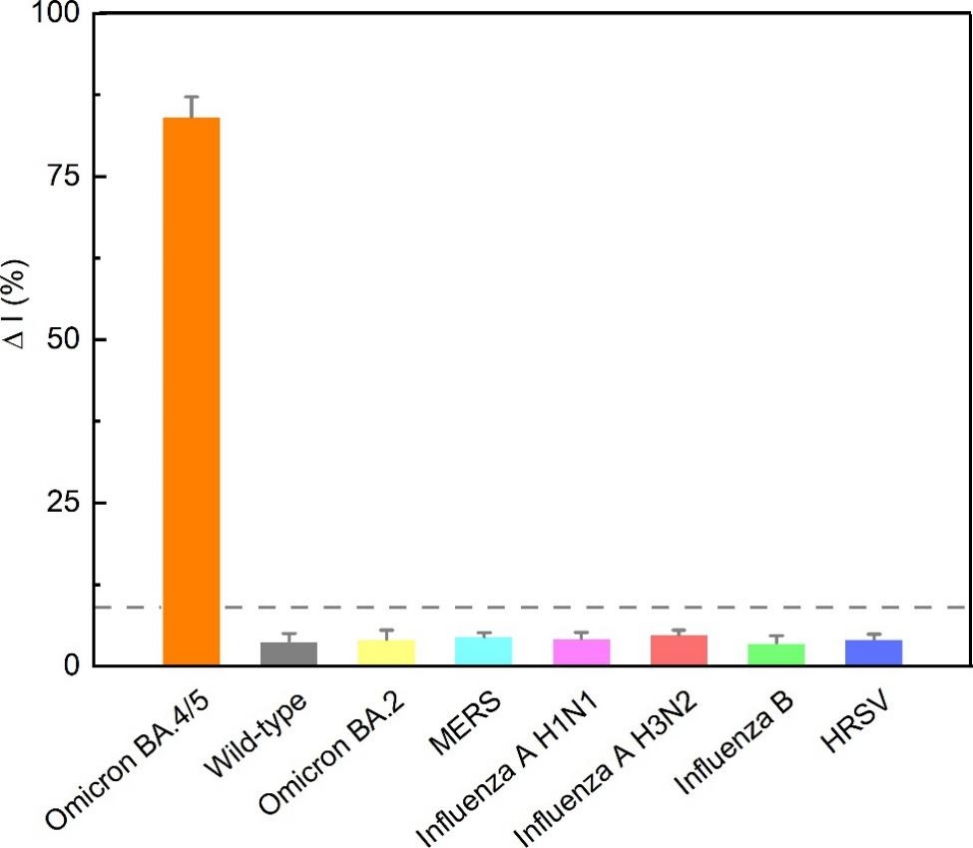
Specificity analysis of the MXene-AuNPs assisted MoECS biosensor for Omicron BA.5 variant The intensity was calculated based on the square wave voltammetry current with the addition of the target BA. 5 variant RNA (1 nM) and RNA templates from different viruses (1 nM). The dash line represents the threshold for a positive signal. Error bars represent standard derivation obtained in three parallel experiments (AuNP, gold nanoparticle; MoECS, Methodology of electrochemical CRISPR sensing)

In addition to the flexible programmability of crRNA and the effective signal amplification efficiency of Cas13a, the high specificity of the Cas13a-crRNA system was another critical advantage. We investigated the specificity of the CRISPR-Cas13a-based electrochemical biosensor using seven related virus/bacterial strains (Table S2). Significant signal changes (△I%–84.1%) was observed towards the target RNA (1 nM) of the Omicron BA. 5 variant. In contrast, △I% was lower than 20% in the detection of interferential parts, which was contiguous to the signal changes of the blank control, indicating the superior selectivity of the fabricated biosensor in differentiating the Omicron BA. 5 variant from nontarget species (Fig. 5). Moreover, the long-term stability of in vitro diagnostic devices is critical for practical applications; thus the stability and reproducibility of MXene-AuNP-assisted MoECS biosensors have been characterized. When the biosensors were stored under nitrogen at 4 °C, the SWV signal showed mineral decrease (< 20%) for up to a week (Figure S7), which is a sufficient turnaround time for the detection of SARS-COV-2 nucleic acid.

**Fig. 6:**
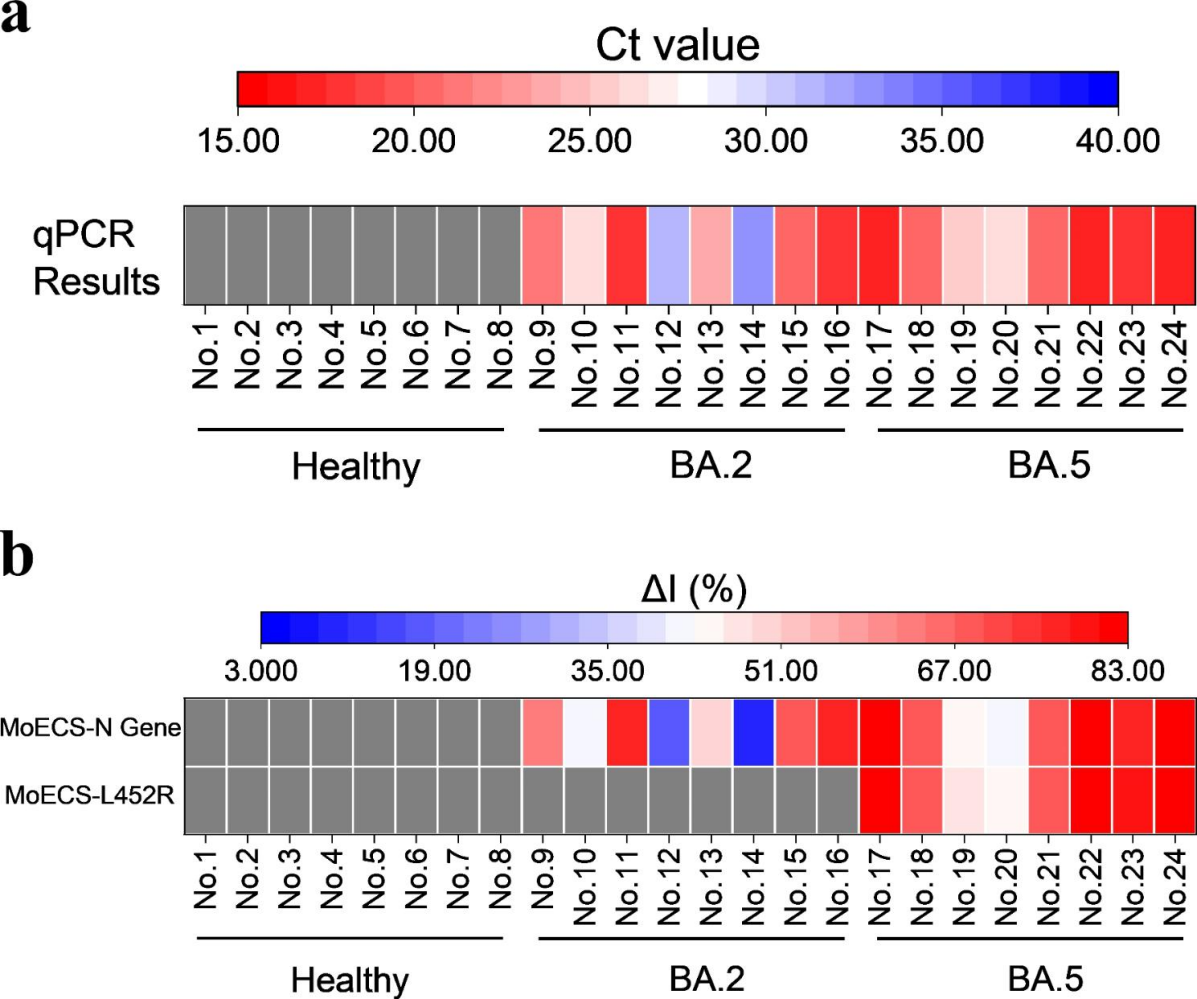
Comparison of the qPCR and MoECS on testing the clinical samples. **(a)** After identifying with qPCR and Sanger sequencing, eight healthy samples, eight BA.2 samples and eight BA.5 samples were categorized. **(b)** MoECS test were performed on the categorized samples, electrodes targeting the N gene showed a highly correlated signal with qPCR results. Moreover, for the electrodes targeting the L452R mutation, signals were only observed in BA.5 samples

Based on the fabrication craft of the electrode and verifications of sensitivity and specificity of CRISPR/Cas13a system for BA.2 or BA.5 variants, practical analysis of clinical samples was performed. From Apr. 2022 to Sep. 2022, sixteen samples from SARS-CoV-2 patients were identified using qPCR and Sanger sequencing by Shenzhen Center for Disease Control and Prevention (China). During the period of sampling, only BA.2 and BA.5 variants were found. Eight samples from healthy people were also used as the negative control. qPCR results (Fig. 6) for the 16 patients were all positive (Ct value varied from 17 to 33), and the results for 8 healthy people were all negative. MoECS test were performed on the categorized samples, and electrodes targeting the N gene showed a highly correlated signal with qPCR results. In accordance to the qPCR results, no healthy sample showed positive result. Without the pre-amplification step, 100% of the positive samples were reported by the electrodes testing N sequence (common qPCR testing site) of the samples. Furthermore, detection besed on MoECS platform for the L452R mutation site (only exists in the BA.5 variant but not in BA.2 variant) was performed using the same samples. The positive result had only shown in the samples of BA.5. These results demonstrated that positive results and the vital L452R variant of SARS-Cov-2 can be reported by MoECS platform simultaneously. Above all, MoECS platform.

## Conclusions

Herein, we propose AuNP-functionalized MXene hybrids using a one-step in situ wet-chemical method and its novel applications in MoECS for the highly specific and sensitive detection of Omicron BA.5 variants of SARS-CoV-2. Since BA.5 variants are more infectious and tolerant to the current vaccines, a rapid, sensitive, and specific method to detect them in patients in the early stage is crucial for the fight against SARS-CoV-2. In this study, MXene-AuNPs improved the sensing performance by increasing conductivity and the electrochemically active surface area. Because of the high specificity of the Cas13a-crRNA system, RNA samples with a single nucleotide polymorphism site were accurately distinguished, and no PAM sequence was required, making the system flexible and programmable. Furthermore, this system showed a wide linear range from 1 nM to 10 fM with high linearity (R^2^ = 0.984) and achieved an ultralow LoD of 1 fM, providing high sensitivity for direct RNA detection from SARS-CoV-2 sequences without the need for pre-amplification. The detection was completed in 1 h, and the biosensor displayed ultra-high specificity and long-term stability. However, our study also has some limitations that need to be addressed in future research. First, our biosensor has not been tested with other variants of SARS-CoV-2 that may have different mutations or characteristics. Secondly, our biosensor has not been validated with a large number of clinical samples from different sources and locations. In summary, This CRISPR-Cas13a-based electrochemical biosensor will be profoundly significant for the early diagnosis of SARS-CoV-2 Omicron BA.5 in a pandemic, compared to current RT-qPCR method. It can also be applied for the detection of other SARS-CoV-2 variants and other pathogens in the future.

## Experimental section

### Materials and instruments

Sodium hydroxide (NaOH), sulfuric acid (H_2_SO_4_), potassium chloride (KCl), sodium chloride (NaCl), magnesium chloride (MgCl_2_), 6-mercaptoethanol (MCH), hydrofluoric acid (HF), dimethyl sulfoxide (DMSO), chloroauric acid (HAuCl_4_), K_3_[Fe(CN)_6_], sodium citrate dihydrate (C_6_H_5_Na_3_O_7_·2H_2_O), K_4_[Fe(CN)_6_], Tris–HCl, Tris-(2-carboxyethyl) phosphine hydrochloride (TCEP), and ethylenediaminetetraacetic acid (EDTA) (all in analytical grade) were obtained from Macklin Biochemical Co., Ltd. (Shanghai, China). Ti_3_AlC_2_ powder was purchased from Xianfeng Nanomaterial Technology Co., Ltd. (Nanjing, China). Cas13a protein and 10x Cas13a Reaction Mix were obtained from EZassay Biotech (Shenzhen, China). Primers were synthesized by Shanghai Generay Biotech Co., Ltd. (Shanghai, China). Murine RNase inhibitor (40 U/µL), DNase I, HiScribe™ T7 High Yield RNA Synthesis Kit, and Monarch RNA Cleanup Kit, were purchased from New England Biolabs Inc. (Ipswich, MA, USA). FAM-ssRNA reporter, crRNA, and MB-ssRNA reporter were synthesized by Sangon Biotech (Shanghai) Co., Ltd. (Shanghai, China) and purified using PAGE.

CHI 760E electrochemical workstation (Shanghai Chenhua Instrument Co., Ltd., China) was performed with electrochemical measurements. The three-electrode system, include working (AuE, 3 mm in diameter), reference (Ag/AgCl), and counter (platinum, Pt wire) electrodes. The morphology and EDS mapping of MXene and MXene-AuNPs were characterized by SEM (TESCAN MIRA LMS).

DNA concentrations were calculated using a NanoDrop 1000 spectrophotometer (Thermo Fisher Scientific, USA). A Cary Eclipse fluorescence spectrophotometer (Agilent Technologies, Palo Alto, CA) was used for the fluorescence spectra reading. An electrophoresis analyzer (Bio-Rad, USA) and a ChemiDoc XRS system (Bio-Rad, USA) were used for agarose gel electrophoresis analysis.

### Preparation of Ti_3_C_2_ MXene nanosheets

Ti_3_C_2_ MXene nanosheets were prepared by etching Ti_3_AlC_2_ with HF. Briefly, 0.1 g Ti_3_AlC_2_ powder was dispersed in 2 mL HF aqueous solution (40%) slowly with continuous stirring at 45 °C for 24 h to gain a stable suspension. The final black suspension was centrifuged (5000 rpm, 15 min) and repeatedly washed to remove any residual HF (final pH ≥ 6). The HF-etched 0.05 g Ti_3_C_2_ powder was then mixed with 1 mL DMSO and continuously stirred for 24 h to obtain a stable suspension. The resultant suspension was then centrifuged (4000 rpm, 5 min) and repeatedly washed to collect the sediment. The sediment was again dispersed in 6 mL of deionized water and sonicated in ice water for 1 h, accompanied with nitrogen bubbling. Finally, the solution was centrifuged (3000 rpm, 30 min), and the dark Ti_3_C_2_ colloidal solution was collected for further experiments.

### Preparation of MXene-AuNPs composites

First, 50 µL of sodium citrate solution (40 mM) was added to 100 µL of HAuCl_4_ (25 mM) and mixed thoroughly to obtain a HAuCl_4_-citrate solution. Subsequently, the HAuCl_4_-citrate solution was slowly dropped into a colloidal solution of MXene (100 µL, 1 mg/mL) and sonicated for 5 min for complete response. After allowing the reaction for another 30 min, the mixed solution was centrifuged (7000 rpm, 7 min) and washed with deionized water to remove any residual reactants. The final volume of the MXene–AuNP solution was fixed at 150 µL for further use and characterization.

### Preparations of SARS-CoV-2 nucleic acid fragments

Specific crRNAs were synthesized, as shown in Table S1. Cas13a crRNA contains two functional parts: one is the scaffold region (GAUUUAGACUACCCCAAAAACGAAGGGGACUAAAAC) for recognization and binding of LbCas13a protein, and the other one is a customized region, which was a reverse complement of the target sequence (~ 28 nt), extending from the 3′ ends of the scaffold to ensure specificity. The SNP site was set at the third base in the customized region, and an additional mismatch base was introduced at the fifth base in this region to achieve the greatest difference in the cleavage between on-target and off-target sequences. L452R (24,410 G ˃ A) is a featured mutation found only in the BA.5 Omicron variants and was used to discern BA.5 and BA.2 variants. The target RNA sequence for L452R detection on the spike (S) gene is listed in Table S2. The RNA templates from respiratory pathogens between T7-promotor and terminator in pUC57 plasmids (Table S2) were synthesized using HiScribe™ T7 High Yield RNA Synthesis Kit, and DNA templates were removed using DNase I. Finally, the RNA templates were purified using the Monarch RNA Cleanup Kit, and the concentration was determined using NanoDrop (Thermo Fisher Scientific) and stored at − 80 °C until use. All the plasmids and crRNAs were provided by Sangon Biotech Co., Ltd. (Shanghai, China). A viral RNA extraction kit (4,992,285, TIANGEN, Beijing, China) was used to prepare RNAs from SARS-CoV-2 wild-type, BA.2, and BA.5 pseudovirus (L02087A, L02087-029, and L02087-031, respectively; Genscript, Nanjing, China) according to the manufacturer’s instructions.

### Fabrication of the ssRNA-Modified electrode

Firstly, the bare AuE was carefully polished using alumina powder (0.3 μm and 0.05 μm in diameter) to obtain a mirror-like surface. It was then sonicated with absolute ethanol and deionized water. Oxides and impurities were removed from the AuE surface by electrochemical cleaning. Next, a series of CV cycles were carried out with the scan rate of 0.1 V/s, and the potential range from − 1 V to 1 V in 0.5 M NaOH, 0.5 M H_2_SO_4_, and 0.1 M H_2_SO_4_ with 0.01 M KCl, respectively. After that, the AuE was washed with deionized water and dried with nitrogen for further use. The MXene-AuNPs modified AuE (AuE-MXene-AuNPs) was fabricated by dropping pre-prepared MXene-AuNPs solution (6 µL) onto the AuE surface and dried in a nitrogen atmosphere for further use [45].

For sample analysis, thiolated MB-ssRNA reporter (100 µM) was pretreated with 10 mM TCEP in the dark at 37 °C for two hours to reduce the S–S bonds [41]. Next, the reduced MB-ssRNA was diluted by Tris-buffer solution (10 mM Tris–HCl, 2 mM EDTA, 10 mM MgCl_2_, 0.1 M NaCl) to 1 µM. The diluted MB-ssRNA reporter (10 µL) was directly dropped onto the surface of the as-prepared AuE-MXene-AuNP and incubated for four hours in the dark at 37 °C and 100% humidity (Figure S8). The electrode was rinsed with Tris-HCl buffer and then immersed in Tris-HCl buffer with 1 mM MCH for 1 h. After thorough washing with Tris-HCl buffer, the electrode was dried with nitrogen for further use. Note that the pH value and concentration of the buffer solution are 7.4 and 10 mM, respectively. The modified AuE can be short-term stored at 4 °C under nitrogen protection in the dark.

### Electrochemical detection

In the electrochemical detection of the trans-cleaved ssRNA, a Cas13a-assisted cleavage assay was performed by mixing Cas13a-crRNA duplex (50 nM Cas13a, 50 nM crRNA, 1× Cas13a Reaction Buffer containing 0.1 U/µL Murine RNase Inhibitor) with the target RNA from BA. 5 pseudovirus to form the Cas13a-crRNA-target RNA triplex. The triplex was then dropped onto the ssRNA-modified electrode and incubated at 37 °C for 45 min under humid conditions. Following the Cas13a-assisted cleavage assay, the electrodes were rinsed with Tris-HCl buffer thoroughly and dried with nitrogen before the subsequent electrochemical analysis.

Electrochemical SWV (frequency of 25 Hz, modulation amplitude of 25 mV, potential increment of 4 mV, and potential ranging from − 0.45 to 0 V vs. Ag/AgCl) was carried out in 10 mM Tris-HCl buffer (pH 7.4) containing 0.1 M NaCl. The △I (%) was calculated as $\frac{{\text{I}}_{0}-\text{I}}{{\text{I}}_{0}}$, where I_0_ and I represent the current before (without target RNA) and after (with target RNA) cleavage, respectively. The (Electrochemical Impedance Spectroscopy) EIS was carried out in 0.1 M KCl solution containing 5 mM [Fe(CN)_6_]^3−/4−^, 5 mV amplitude, the biased potential of 0.23 V (vs. Ag/AgCl) in the frequency range of 0.01–100,000 Hz.

### Analysis of mismatch in RNA samples

The specificity of each designed crRNA targeting RNA sample was validated by a CRISPR-Cas13a diagnosis assay based on fluorescence signals. The reaction mix contained 50 nM Cas13a (NEB), 50 nM crRNA, 1× Cas13a reaction buffer, 500 nM of single-stranded RNA (ssRNA) reporter (5′ 6-FAM/UUUUU/BHQ-1 3′, Sangon), 0.1 U/µL murine RNase inhibitor, RNA templates from pseudovirus of wild-type, BA.2, or BA.5 gene sequences, and DNA sequences from plasmids containing influenza virus, MERS, and HRSV sequences dispersed in pure water. During the incubation at 37 °C for 30 min, real-time fluorescence was measured with an excitation wavelength of 485 nm and emission wavelength of 528 nm. The trans- and cis-cleavage were further verified by electrophoresis (2.5% agarose gel, 100 V at 4 ℃ for 60 min) and observed using an electrophoresis analyzer.

### Clinical samples

To validate the clinical efficacy of this sensing system, 24 clinical samples were by nasopharyngeal swab, including sixteen SARS-CoV-2 positive samples and eight healthy samples, which were already determined by qPCR and Sanger sequencing. These samples were provided under the ethics approval issued by Shenzhen Center for Disease Control and Prevention (China), approval No. is SJLZ [2022] No. 07 A. The clinical samples were inactivated for 30 min at 56 ℃, and the released RNA was tested.

### Statistics

The data are displayed as mean ± standard deviation, all measurements were performed in three parallel experiments (n = 3). Correlations were performed with linear regression to determine the goodness of fit (Pearson’s correlation coefficient, R2). For inter-sample comparisons, multiple pairs of samples were analyzed by a two-tailed t-test, and the resulting P values were adjusted for multiple hypothesis testing using Bonferroni correction. *P < 0.05, **P < 0.01, and ***P < 0.001 indicate obvious statistical differences. All statistical analyses were performed using OriginPro (v.2022).

## Electronic supplementary material

Below is the link to the electronic supplementary material.

Supplementary Material 1

## Data Availability

The datasets used and analyzed during the current study are available from the corresponding author upon reasonable request.
