# Supplementary material for: CRISPR-Cas13a-powered electrochemical biosensor for the detection of the L452R mutation in clinical samples of SARS-CoV-2 variants
Source: J Nanobiotechnology. 2023 Apr 29;21:141. doi: 10.1186/s12951-023-01903-5 (PMC10148006; doi:10.1186/s12951-023-01903-5)
Supplement: Supplementary file 1 — Supplementary Material 1 [file 12951_2023_1903_MOESM1_ESM.docx]

**CRISPR-Cas13a-powered electrochemical biosensor for the detection of the L452R mutation in clinical samples of SARS-CoV-2 variants**

***Supplementary Information***

Zhi Chen ^1,2,#^, Chenshuo Wu ^2,3,#^, Yuxuan Yuan ^2^, Zhongjian Xie ^4^, Tianzhong Li ^2^, Hao Huang ^1^, Shuang Li ^5^, Jiefeng Deng ^5^, Huiling Lin ^6^, Zhe Shi ^7^, Chaozhou Li ^2^, Yabin Hao ^2^, Yuxuan Tang ^8^, Yuehua You ^9,10^, Omar A. Al-Hartomy ^11^,

Swelm Wageh ^11^, Abdullah G. Al-Sehemi ^12,13^, Ruitao Lu ^18^, Ling Zhang ^14^, Xuechun Lin ^15^, Yaqing He ^16,*^, Guojun Zhao ^1,*^, Defa Li ^17,*^, and Han Zhang ^2,*^

1 The Sixth Affiliated Hospital of Guangzhou Medical University, Qingyuan People's Hospital, Qingyuan 511518, Guangdong, People's Republic of China.

2 International Collaborative Laboratory of 2D, Materials for Optoelectronics Science and Technology of Ministry of Education, Institute of Microscale Optoelectronics, College of Physics and Optoelectronic Engineering, Shenzhen University, Shenzhen 518060, People's Republic of China.

3 Yangtze Delta Region Institute (Huzhou), University of Electronic Science and Technology of China, Huzhou, 313001, People's Republic of China.

4 Institute of Pediatrics, Shenzhen Children's Hospital, Institute of Pediatrics, Shenzhen Children's Hospital, Clinical Medical College of Southern University of Science and Technology, Shenzhen 518038, Guangdong, P. R. China

5 College of Pharmacy, Dali University, Dali 671000, P. R. China

6 Hengyang Medical College, University of South China, Hengyang, Hunan 421001, China

7 School of Physics & New Energy, Xuzhou University of Technology, Xuzhou, Jiangsu, 221018, People's Republic of China.

8 Shenzhen Metasensing Tech Limited Company, Shenzhen 518000, People's Republic of China.

9 Department of Stomatology, Longhua People's Hospital Affiliated to Southern Medical University, Shenzhen 518109, People's Republic of China.

10 School of Stomatology, Southern Medical University, Guangzhou 510515, People's Republic of China.

11 Department of Physics, Faculty of Science, King Abdulaziz University, Jeddah 21589, Saudi Arabia

12 Research Center for Advanced Materials Science (RCAMS), King Khalid University, P.O. Box 9004, Abha, 61413, Saudi Arabia

13 Department of Chemistry, College of Science, King Khalid University, P.O. Box 9004, Abha 61413, Saudi Arabia

14 Key Lab of Semiconductor Materials Science, Institute of Semiconductors, Chinese Academy of Sciences, Beijing, 100083

15 Laboratory of All-Solid-State Light Sources, Institute of Semiconductors, Chinese Academy of Sciences, Beijing 100083

16 Shenzhen Center for Disease Control and Prevention, Shenzhen 518055, Guangdong, P. R. China

17 Department of Laboratory Medicine, Shenzhen Children's Hospital, Shenzhen 518038, Guangdong, People's Republic of China.

18 Shenzhen International Institute for Biomedical Research, Shenzhen 518110, China

#Zhi Chen and Chenshuo Wu contributed equally to this work and should be regarded as the co-first authors.

**Corresponding authors: heyaqing1019@126.com, zhaoguojun@gzhmu.edu.cn, dfl_szetyy@126.com, and hzhang@szu.edu.cn**

**Figure S1** XRD patterns of Ti_3_AlC_2_ and Ti_3_C_2_T_x_ powders.


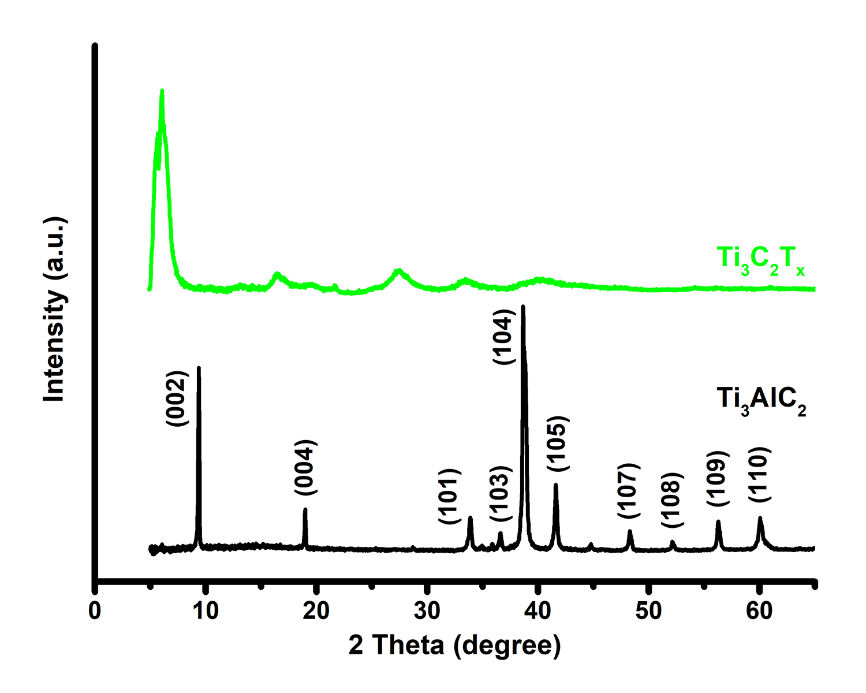


**Figure S2** Different magnification SEM images of MXene-AuNPs modified AuE.


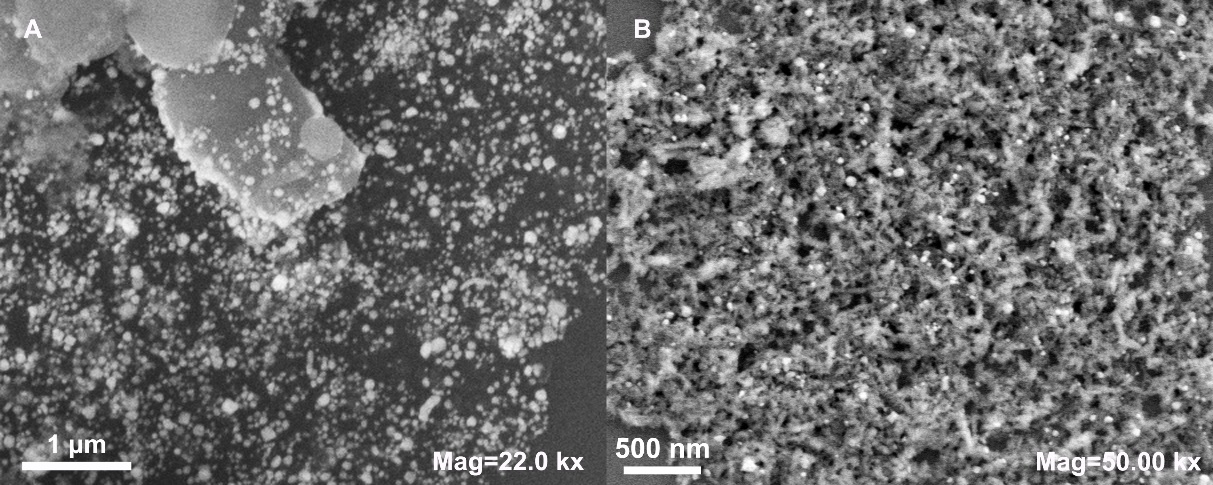


**Figure S3** High frequency region of EIS of bare AuE and MXene-AuNPs modified AuE.


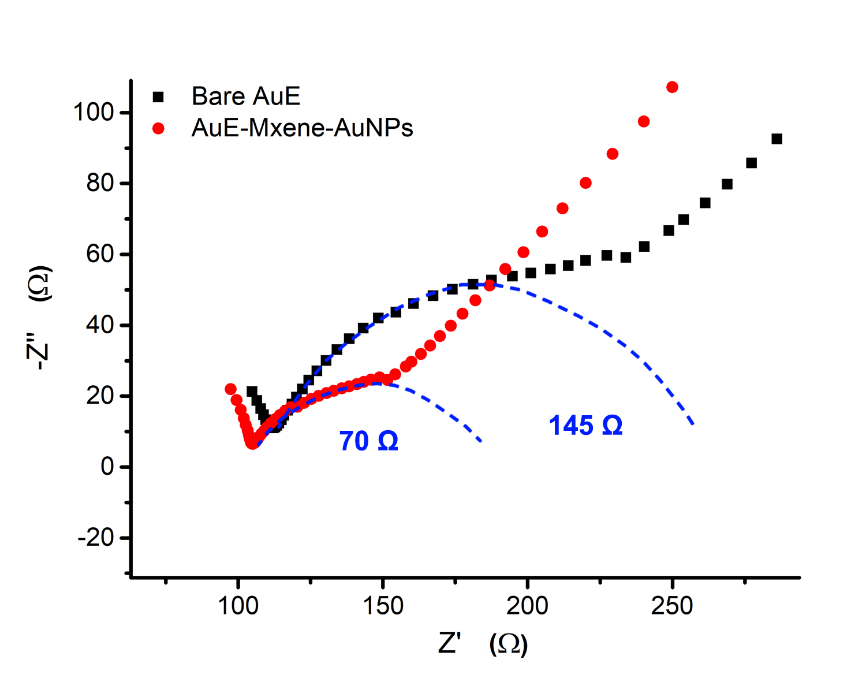


EIS was performed in 0.1 M KCl solution contenting 5 mM [Fe(CN)_6_]^3-/4-^, biased potential of 0.23 V (vs. Ag/AgCl) in the frequency range of 0.01–10^5^ Hz, and 5 mV amplitude.

**Figure S4** CV curves of MXene nanosheets modified electrode and MXene-AuNPs modified electrode.


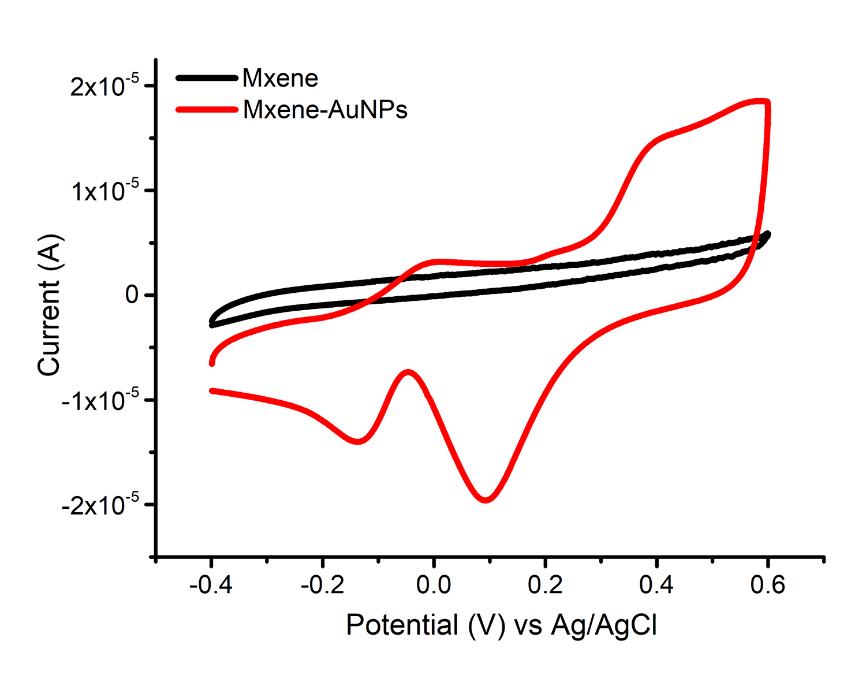


The CV was carried out in 0.5 M KOH with a potential range from -0.4 to 0.6 V vs Ag/AgCl at the scan rate of 100 mV/s.

**Figure S5** Validation of trans- and cis-cleavage through agarose gel electrophoresis.


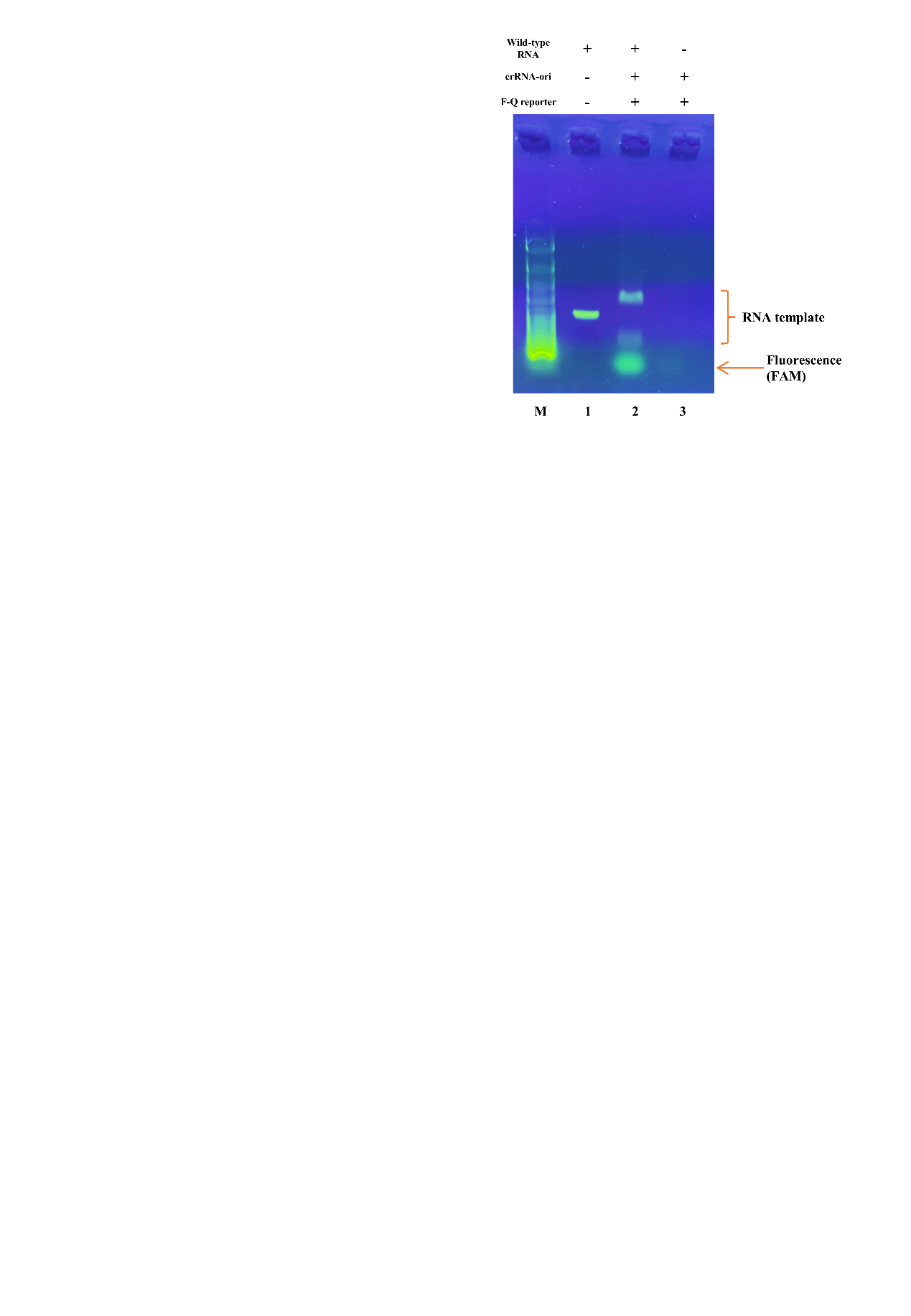


**Figure S6** Correlations between Fluorescence intensity and L452R RNA template ranged from 10 fM-10 nM performed with SHERLOCK assay. The limit of detection was calculated to be 1.72 pM.


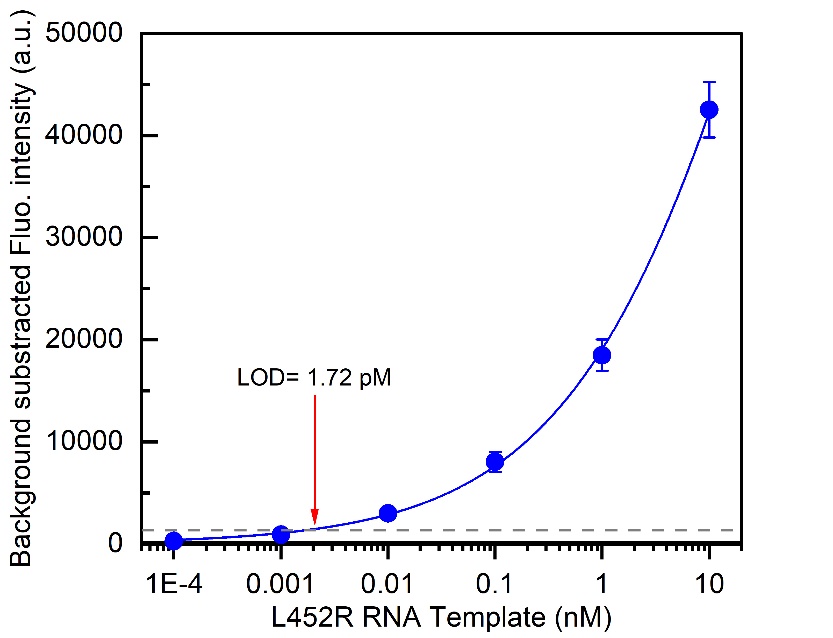


**Figure S7** Evaluation of the storage ability of the optimized ssRNA electrode surface. 100% signal was obtained through SWV test on the freshly prepared ssRNA electrode. The prepared ssRNA covered sensors were stored at 4 °C in the dark under nitrogen protection. Error bars represent standard derivation obtained in three parallel experiments.


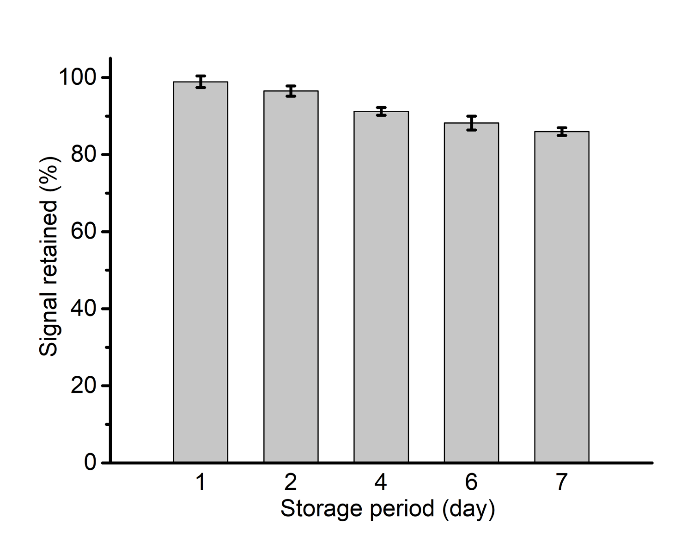


**Figure S8** The electrochemical signal of MB-ssRNA at different immobilization times ranging from 1 to 6 hours.


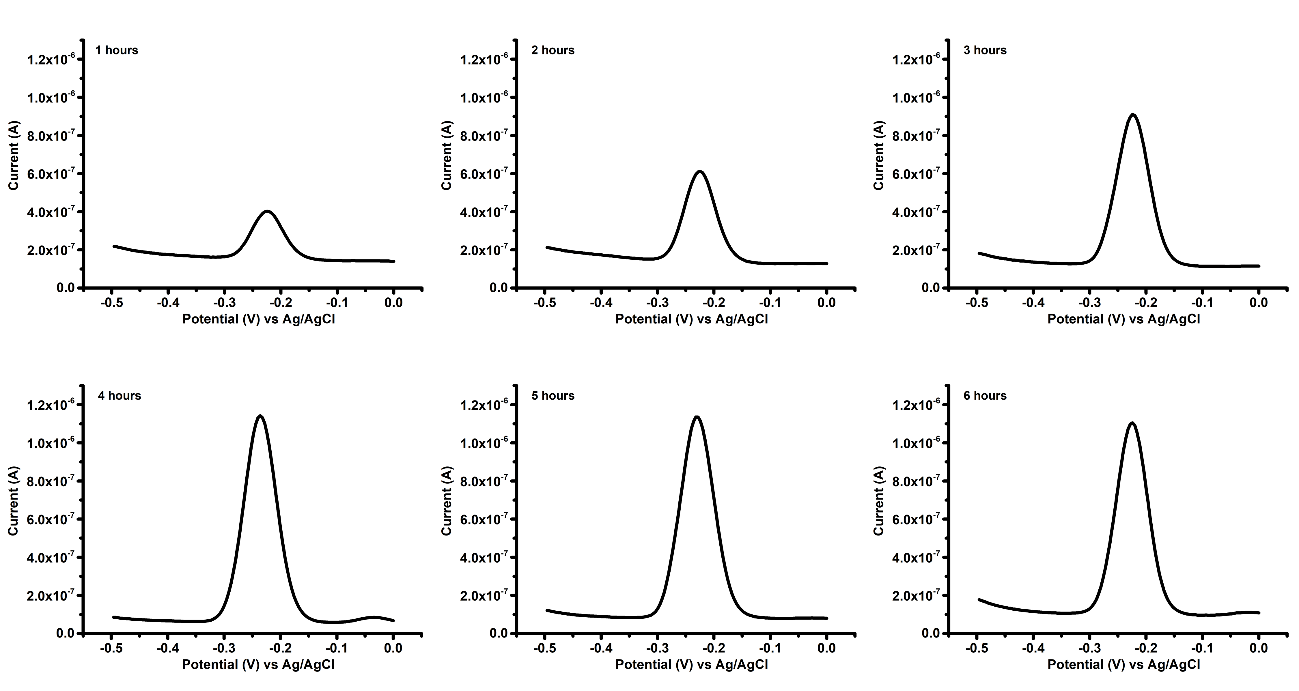


**Table S1** CRISRP-Cas13a-crRNA and ssRNA reporters used in this study. crRNA was designed according to the “CRISPR-SHERLOCK” method. Lower-case letters indicate the “scaffold” sequence for LbCas13a protein. Base labelled in RED indicate the single nucleotide polymorphism (SNP) site; base labelled in BLUE indicate the “deliberately” introduced mismatch.

| Name | Sequence (5’-3’) |
| --- | --- |
| crRNA-ori | gauuuagacuaccccaaaaacgaaggggacuaaaacACAGGUAAUUAUAAUUACCACCAACCUU |
| crRNA-mis1 | gauuuagacuaccccaaaaacgaaggggacuaaaacGCAGGUAAUUAUAAUUACCACCAACCUU |
| crRNA-mis3 | gauuuagacuaccccaaaaacgaaggggacuaaaacACCGGUAAUUAUAAUUACCACCAACCUU |
| crRNA-mis5 | gauuuagacuaccccaaaaacgaaggggacuaaaacACAGAUAAUUAUAAUUACCACCAACCUU |
| crRNA-mis7 | gauuuagacuaccccaaaaacgaaggggacuaaaacACAGGUGAUUAUAAUUACCACCAACCUU |
| crRNA-mis9 | gauuuagacuaccccaaaaacgaaggggacuaaaacACAGGUAAGUAUAAUUACCACCAACCUU |
| crRNA-mis11 | gauuuagacuaccccaaaaacgaaggggacuaaaacACAGGUAAUUGUAAUUACCACCAACCUU |
| crRNA-mis13 | gauuuagacuaccccaaaaacgaaggggacuaaaacACAGGUAAUUAUGAUUACCACCAACCUU |
| crRNA-mis15 | gauuuagacuaccccaaaaacgaaggggacuaaaacACAGGUAAUUAUAAGUACCACCAACCUU |
| crRNA-mis3+1 | gauuuagacuaccccaaaaacgaaggggacuaaaacGCCGGUAAUUAUAAUUACCACCAACCUU |
| crRNA-mis3+2 | gauuuagacuaccccaaaaacgaaggggacuaaaacAACGGUAAUUAUAAUUACCACCAACCUU |
| crRNA-mis3+4 | gauuuagacuaccccaaaaacgaaggggacuaaaacACCCGUAAUUAUAAUUACCACCAACCUU |
| crRNA-mis3+5 | gauuuagacuaccccaaaaacgaaggggacuaaaacACCGUUAAUUAUAAUUACCACCAACCUU |
| crRNA-mis3+6 | gauuuagacuaccccaaaaacgaaggggacuaaaacACCGGGAAUUAUAAUUACCACCAACCUU |
| crRNA-mis3+7 | gauuuagacuaccccaaaaacgaaggggacuaaaacACCGGUUAUUAUAAUUACCACCAACCUU |
| crRNA-mis3+8 | gauuuagacuaccccaaaaacgaaggggacuaaaacACCGGUAUUUAUAAUUACCACCAACCUU |
| crRNA-mis3+9 | gauuuagacuaccccaaaaacgaaggggacuaaaacACCGGUAAGUAUAAUUACCACCAACCUU |
| MB-ssRNA reporter | SH-UUUUUUUUUU-MB |
| crRNA-N gene | gauuuagacuaccccaaaaacgaaggggacuaaaacCCCCCAGCGCUUCAGCGUUC |
| FAM-ssRNA reporter | FAM-UUUUU-BHQ1 |

**Table S2** Sequences from SARS-CoV-2 (wild-type, BA.4/5, or BA.2), Middle East respiratory syndrome-related coronavirus (MERS), Influenza A virus (H1N1/H3N2), Influenza B virus, Human Respiratory Syncytial Virus (HRSV). These sequences were inserted into pUC57 plasmids between T7 promoter sequence (TAATACGACTCACTATAGGG) and terminator sequence (TTCCATCTGTTTTCTTATCTGTTCTTTCATCTGTTCTTTATCTGTTTGTTT). The mutation site labelled in RED indicates the specific mutation of L452R in BA4/5 variant (L452R: 22917 T ˃ G), the mutation sites labelled in GREEN indicate mutations both in BA.2 and BA.4/5 variant (D405N, R408S, K417N, N440K, S477N, T478K, E484A, Q493R, Q498R, N501Y, Y505H, respectively).

| Name | Inserted Sequences (5’-3’) |
| --- | --- |
| SARS-CoV-2  wild-type  (NC_045512.2, S gene, 22736-23080) | TTTACTAATGTCTATGCAGATTCATTTGTAATTAGAGGTGATGAAGTCAGACAAATCGCTCCAGGGCAAACTGGAAAGATTGCTGATTATAATTATAAATTACCAGATGATTTTACAGGCTGCGTTATAGCTTGGAATTCTAACAATCTTGATTCTAAGGTTGGTGGTAATTATAATTACCTGTATAGATTGTTTAGGAAGTCTAATCTCAAACCTTTTGAGAGAGATATTTCAACTGAAATCTATCAGGCCGGTAGCACACCTTGTAATGGTGTTGAAGGTTTTAATTGTTACTTTCCTTTACAATCATATGGTTTCCAACCCACTAATGGTGTTGGTTACCAA |
| SARS-CoV-2  BA.2 | TTTACTAATGTCTATGCAGATTCATTTGTAATTAGAGGTAATGAAGTCAGACAAATCGCTCCAGGGCAAACTGGAAATATTGCTGATTATAATTATAAATTACCAGATGATTTTACAGGCTGCGTTATAGCTTGGAATTCTAACAAGCTTGATTCTAAGGTTGGTGGTAATTATAATTACCTGTATAGATTGTTTAGGAAGTCTAATCTCAAACCTTTTGAGAGAGATATTTCAACTGAAATCTATCAGGCCGGTAACAAACCTTGTAATGGTGTTGCAGGGTTTAATTGTTACTTTCCTTTACGATCATATGGTTTCCGACCCACTTATGGTGTTGGTCACCAA |
| SARS-CoV-2 BA.4/5 | TTTACTAATGTCTATGCAGATTCATTTGTAATTAGAGGTAATGAAGTCAGACAAATCGCTCCAGGGCAAACTGGAAATATTGCTGATTATAATTATAAATTACCAGATGATTTTACAGGCTGCGTTATAGCTTGGAATTCTAACAAGCTTGATTCTAAGGTTGGTGGTAATTATAATTACCGGTATAGATTGTTTAGGAAGTCTAATCTCAAACCTTTTGAGAGAGATATTTCAACTGAAATCTATCAGGCCGGTAACAAACCTTGTAATGGTGTTGCAGGGTTTAATTGTTACTTTCCTTTACGATCATATGGTTTCCGACCCACTTATGGTGTTGGTCACCAA |
| MERS (NC_019843.3, 24275-24938) | TGTTAATATGGAAGCCGCGTATACTTCATCTTTGCTTGGCAGCATAGCAGGTGTTGGCTGGACTGCTGGCTTATCCTCCTTTGCTGCTATTCCATTTGCACAGAGTATCTTTTATAGGTTAAACGGTGTTGGCATTACTCAACAGGTTCTTTCAGAGAACCAAAAGCTTATTGCCAATAAGTTTAATCAGGCTCTGGGAGCTATGCAAACAGGCTTCACTACAACTAATGAAGCTTTTCAGAAGGTTCAGGATGCTGTGAACAACAATGCACAGGCTCTATCCAAATTAGCTAGCGAGCTATCTAATACTTTTGGTGCTATTTCCGCCTCTATTGGAGACATCATACAACGTCTTGATGTTCTCGAACAGGACGCCCAAATAGACAGACTTATTAATGGCCGTTTGACAACACTAAATGCTTTTGTTGCACAGCAGCTTGTTCGTTCCGAATCAGCTGCTCTTTCCGCTCAATTGGCTAAAGATAAAGTCAATGAGTGTGTCAAGGCACAATCCAAGCGTTCTGGATTTTGCGGTCAAGGCACACATATAGTGTCCTTTGTTGTAAATGCCCCTAATGGCCTTTACTTCATGCATGTTGGTTATTACCCTAGCAACCACATTGAGGTTGTTTCTGCTTATGGTCTTTGCGATGCAGCTAACCCT |
| Influenza A virus, H1N1  (NC_026431.1) | ATGAGTCTTCTAACCGAGGTCGAAACGTACGTTCTTTCTATCATCCCGTCAGGCCCCCTCAAAGCCGAGATCGCGCAGAGACTGGAAAGTGTCTTTGCAGGAAAGAACACAGATCTTGAGGCTCTCATGGAATGGCTAAAGACAAGACCAATCTTGTCACCTCTGACTAAGGGAATTTTAGGATTTGTGTTCACGCTCACCGTGCCCAGTGAGCGAGGACTGCAGCGTAGACGCTTTGTCCAAAATGCCCTAAATGGGAATGGGGACCCGAACAACATGGATAGAGCAGTTAAACTATACAAGAAGCTCAAAAGAGAAATAACGTTCCATGGGGCCAAGGAGGTGTCACTAAGCTATTCAACTGGTGCACTTGCCAGTTGCATGGGCCTCATATACAACAGGATGGGAACAGTGACCACAGAAGCTGCTTTTGGTCTAGTGTGTGCCACTTGTGAACAGATTGCTGATTCACAGCATCGGTCTCACAGACAGATGGCTACTACCACCAATCCACTAATCAGGCATGAAAACAGAATGGTGCTGGCTAGCACTACGGCAAAGGCTATGGAACAGATGGCTGGATCGAGTGAACAGGCAGCGGAGGCCATGGAGGTTGCTAATCAGACTAGGCAGATGGTACATGCAATGAGAACTATTGGGACTCATCCTAGCTCCAGTGCTGGTCTGAAAGATGACCTTCTTGAAAATTTGCAGGCCTACCAGAAGCGAATGGGAGTGCAGATGCAGCGATTCAAGTGATCCTCTCGTCATTGCAGCAAATATCATTGGGATCTTGCACCTGATATTGTGGATTACTGATCGTCTTTTTTTCAAATGTATTTATCGTCGCTTTAAATACGGTTTGAAAAGAGGGCCTTCTACGGAAGGAGTGCCTGAGTCCATGAGGGAAGAATATCAACAGGAACAGCAGAGTGCTGTGGATGTTGACGATGGTCATTTTGTCAACATAGAGCTAGAGTAA |
| Influenza A virus, H3N2  (U51247.1) | ATGAATCCAAATCAAAAGATAATAACAATTGGCTCTGTTTCTCTCACTATTGCCACAATATGCTGCCTTATGCAAATTGCCATCCTGGTAACTACTGTAACATTACATTTCAAGCAATATGAATGCAACTCCCCCCCAAACAACCAAGTAATGCTGTGTGAACCAACAATAATAGAAAGAAACATAACAGAGATAGTGTATCTGACCAACACCACCATAGAGAAAGAAGTATGCCCCAAACTAGCAGAATACAGAAATTGGTCAAAGCCGCAATGTAAAATTACAGGATTTGCACCTTTTTCTAAGGACAATTCAATTCGGCTTTCCGCTGGTGGAGACATTTGGGTGACAAGAGAACCTTATGTGTCATGCGATCCTGGCAAGTGTTATCAATTTGCCCTTGGACAGGGAACAACACTAAACAACAGGCATTCAAATGACACAGTACATGATAGGACCCCTTATCGAACCCTATTGATGAATGAGTTGGGTGTTCCATTTCATTTGGGAACCAAGCAAGTGTGCATAGCATGGTCCAGCTCAAGTTGTCACGATGGAAAAGCATGGCTGCATGTTTGTGTAACTGGGCATGATGAAAATGCAACTGCTAGCTTCATTTACGATGGGAGGCTTGTAGATAGTATTGGTTCATGGTCCAAAAATATCCTCAGGACCCAGGAGTCGGAATGCGTTTGTATCAATGGAACTTGTACAGTAGTAATGACTGATGGAAGTGCTTCAGGAAGAGCTGATACTAAAATACTATTCATTGAAGAGGGGAAAATCGCTCATATTAGCCCATTGTCAGGAAGTGCTCAGCATGTCGAGGAGTGCTCCTGTTATCCTCGATATCCTGGTGTCAGATGTGTCTGCAGAGACAACTGGAAAGGCTCCAATAGGCCCATCGTAGATATAAATGTGAAAGATTATAGCATTGTTTCCAGTTATGTGTGCTCAGGACTTGTTGGAGACACAGCCAGAAAAAACGACAGCTCCAGCAGTAGCTATTGCCGGAATCCTAACAATGAGAAAGGGAGTCATGGAGTGAAAGGCTGGGCCTTTGATGATGGAAATGATGTGTGGATGGGAAGAACGATCAGCGAGAAGTTACGCTCAGGTGATGAAACCTTCAAAGTCATTGGAGGCTGGTCCAAACCTAACTCCAAATTGCAGATAAATAGGCAAGTCATAGTTGACAGAGGTAATAGGTCCGGTTATTCTGGTATTTTCTCTGTTGAAGGCAAAAGCTGCATCAATCGGTGCTTTTATGTGGAGTTGATAAGGGGAAGGAAACAGGAAACTGAAGTCTGGTGGACCTCAAACAGTATTGTTGTGTTTTGTGGCACCTCAGGTACATATGGAACAGGCTCATGGCCTGATGGGGCGGACATCAATCTCATGCCTATATAAA |
| Influenza B virus segement 7 (NC_002210.1) | AGCAGAAGCACGCACTTTCTTAAAATGTCGCTGTTTGGAGACACAATTGCCTACCTGCTTTCACTAATAGAAGATGGAGAAGGCAAAGCAGAACTAGCTGAAAAATTACACTGTTGGTTCGGTGGGAAAGAATTTGACCTAGATTCTGCTTTGGAATGGATAAAAAACAAAAGGTGCCTAACTGATATACAAAAAGCACTAATTGGTGCCTCTATATGCTTTTTAAAACCCAAAGACCAAGAAAGAAAAAGGAGATTCATCACAGAGCCCCTGTCAGGAATGGGAACAACAGCAACAAAGAAGAAAGGCCTAATTCTAGCTGAGAGAAAAATGAGAAGATGTGTAAGCTTTCATGAAGCATTTGAAATAGCAGAAGGCCACGAAAGCTCAGCATTACTATATTGTCTTATGGTCATGTACCTAAACCCTGAAAACTATTCAATGCAAGTAAAACTAGGAACGCTCTGTGCTTTATGCGAGAAACAAGCATCGCACTCGCATAGAGCCCATAGCAGAGCAGCAAGGTCTTCGGTACCTGGAGTAAGACGAGAAATGCAGATGGTTTCAGCTATGAACACAGCAAAGACAATGAATGGAATGGGAAAGGGAGAAGACGTCCAAAAACTAGCAGAAGAGCTGCAAAACAACATTGGAGTGTTGAGATCTCTAGGAGCAAGTCAAAAGAATGGAGAAGGAATTGCCAAAGATGTAATGGAAGTGCTAAAACAGAGCTCTATGGGAAATTCAGCTCTTGTGAGGAAATACTTATAATGCTCGAACCACTTCAGATTCTTTCAATTTGTTCTTTCATTTTATCAGCTCTCCATTTCATGGCTTGGACAATAGGGCATTTGAATCAAATAAAAAGAGGGGTAAACTTGAAAATACAAATAAGGAATCCAAATAAGGAGGCAATAAACAGAGAGGTGTCAATTCTGAGACACAATTACCAAAAGGAAATCCAAGCCAAAGAAACAATGAAGAAAATACTCTCTGACAACATGGAAGTATTGGGTGACCACATAGTAGTTGAAGGGCTTTCAACTGATGAGATAATAAAAATGGGTGAAACAGTTTTGGAGGTGGAAGAATTGCAATGAGCCCAATTTTCACTGTATTTCTTACTATGCATTTAAGCAAATTGTAATCAATGTCAGTGAATAAAACTGGAAAAAGTGCGTTGTTTCTACT |
| HRSV, Human orthopneumovirus Subgroup A, (NC_038235.1) | GGGGCAAATACAAAGATGGCTCTTAGCAAAGTCAAGTTGAATGATACACTCAACAAAGATCAACTTCTGTCATCCAGCAAATACACCATCCAACGGAGCACAGGAGATAGTATTGATACTCCTAATTATGATGTGCAGAAACACATCAATAAGTTATGTGGCATGTTATTAATCACAGAAGATGCTAATCATAAATTCACTGGGTTAATAGGTATGTTATATGCGATGTCTAGGTTAGGAAGAGAAGACACCATAAAAATACTCAGAGATGCGGGATATCATGTAAAAGCAAATGGAGTAGATGTAACAACACATCGTCAAGACATTAATGGAAAAGAAATGAAATTTGAAGTGTTAACATTGGCAAGCTTAACAACTGAAATTCAAATCAACATTGAGATAGAATCTAGAAAATCCTACAAAAAAATGCTAAAAGAAATGGGAGAGGTAGCTCCAGAATACAGGCATGACTCTCCTGATTGTGGGATGATAATATTATGTATAGCAGCATTAGTAATAACTAAATTAGCAGCAGGGGACAGATCTGGTCTTACAGCCGTGATTAGGAGAGCTAATAATGTCCTAAAAAATGAAATGAAACGTTACAAAGGCTTACTACCCAAGGACATAGCCAACAGCTTCTATGAAGTGTTTGAAAAACATCCCCACTTTATAGATGTTTTTGTTCATTTTGGTATAGCACAATCTTCTACCAGAGGTGGCAGTAGAGTTGAAGGGATTTTTGCAGGATTGTTTATGAATGCCTATGGTGCAGGGCAAGTGATGTTACGGTGGGGAGTCTTAGCAAAATCAGTTAAAAATATTATGTTAGGACATGCTAGTGTGCAAGCAGAAATGGAACAAGTTGTTGAGGTTTATGAATATGCCCAAAAATTGGGTGGTGAAGCAGGATTCTACCATATATTGAACAACCCAAAAGCATCATTATTATCTTTGACTCAATTTCCTCACTTCTCCAGTGTAGTATTAGGCAATGCTGCTGGCCTAGGCATAATGGGAGAGTACAGAGGTACACCGAGGAATCAAGATCTATATGATGCAGCAAAGGCATATGCTGAACAACTCAAAGAAAATGGTGTGATTAACTACAGTGTACTAGACTTGACAGCAGAAGAACTAGAGGCTATCAAACATCAGCTTAATCCAAAAGATAATGATGTAGAGCTTTGAGTTAATAAAAAA |

**Table S3** **Some most recent and relevant papers about different types of electrochemical biosensors for Covid-19 detection.**

| **Authors** | **Biosensor type** | **Pre-amplification (Nucleic acid only)** | **Target** | **Linear range** | **LOD** | **Detection time** | **Reference** |
| --- | --- | --- | --- | --- | --- | --- | --- |
| Tripathy et al. | LAMP-combined Electrochemistry | LAMP | SARS-CoV-2 Nucleic acid | / | 2.5-25 copies/μL | > 80 min | [1]  (2023) |
| Yang et al. | Electrochemistry using AuNPs-rGO electrodeposited acupuncture needles | /  (Protein) | SARS-CoV-2 spike protein | 0.1 ∼ 1000 ng /mL | 38 pg/mL | 30 min | [2]  (2023) |
| Alafeef et al. | DNA probes immobilized Electrochemical sensor | No need | SARS-CoV-2 N-gene | 50∼2200 copies/μL | 2.5 copies/μL | 5 min | [3]  (2023) |
| Zhou et al. | DSN Based Electrochemical Biosensor | Duplex-specific nuclease | SARS-CoV-2 RdRp gene | 0∼400 aM | 21.69 aM. | >120 min | [4]  (2023) |
| Shi et al. | Electrochemical sensor with CRISPR/Cas12a system and strand displacement reaction | Strand displacement reaction | Strand displacement reaction-dealt DNA sequence of S and Orf1ab genes | 100 aM∼10 pM | 40 aM | > 130 min | [5]  (2023) |
| Kashefi-Kheyrabadi et al. | Electrochemical sensor with CRISPR/Cas13a system | No need | SARS-CoV-2 S and Orf1ab genes | 10^−17^ ∼ 10^−11^ M | 26∼53 copies/μL | 3h | [6]  (2023) |
| Chen et al. | MXene-AuNP enhanced Electrochemical sensor with CRISPR/Cas13a system | No need | SARS-CoV-2 S gene, RNA sequence, **SNP site** | 10fM ∼1nM | 1fM | 45 min | This work |

**References**

1. Tripathy S, Agarkar T, Talukdar A, Sengupta M, Kumar A, Ghosh S: **Evaluation of indirect sequence-specific magneto-extraction-aided LAMP for fluorescence and electrochemical SARS-CoV-2 nucleic acid detection.** *Talanta* 2023, **252:**123809.

2. Yang X, Yin ZZ, Zheng G, Zhou M, Zhang H, Li J, Cai W, Kong Y: **Molecularly imprinted miniature electrochemical biosensor for SARS-CoV-2 spike protein based on Au nanoparticles and reduced graphene oxide modified acupuncture needle.** *Bioelectrochemistry* 2023, **151:**108375.

3. Alafeef M, Skrodzki D, Moitra P, Gunaseelan N, Pan D: **Binding-Induced Folding of DNA Oligonucleotides Targeted to the Nucleocapsid Gene Enables Electrochemical Sensing of SARS-CoV-2.** *ACS Appl Bio Mater* 2023, **6:**1133-1145.

4. Zhou K, Dai J: **A duplex-specific nuclease based electrochemical biosensor for the assay of SARS-CoV-2 RdRp RNA.** *Anal Biochem* 2023, **661:**114983.

5. Shi K, Yi Z, Han Y, Chen J, Hu Y, Cheng Y, Liu S, Wang W, Song J: **PAM-free cascaded strand displacement coupled with CRISPR-Cas12a for amplified electrochemical detection of SARS-CoV-2 RNA.** *Anal Biochem* 2023, **664:**115046.

6. Kashefi-Kheyrabadi L, Nguyen HV, Go A, Lee MH: **Ultrasensitive and amplification-free detection of SARS-CoV-2 RNA using an electrochemical biosensor powered by CRISPR/Cas13a.** *Bioelectrochemistry* 2023, **150:**108364.
